# Elementary school teachers knowledge and attitude towards attention deficit-hyperactivity disorder in Gondar, Ethiopia: a multi-institutional study

**DOI:** 10.1186/s13034-021-00371-9

**Published:** 2021-04-07

**Authors:** Mekdes Dessie, Masresha Asmare Techane, Bizuneh Tesfaye, Daniel Ayelegne Gebeyehu

**Affiliations:** 1 Department of Nursing, Blue Nile Health Science College, Gondar, Ethiopia; 2 Department of Pediatrics and Child Health Nursing, School of Nursing, College of Medicine and Health Sciences, University of Gondar, Gondar, Ethiopia; 3 Department of Psychiatry, School of Medicine, College of Medicine and Health Sciences, University of Gondar, Gondar, Ethiopia; 4 Community Health Nursing Unit, School of Nursing, College of Medicine and Health Sciences, University of Gondar, Gondar, Ethiopia

**Keywords:** Knowledge, Attitude, ADHD, Elementary school teacher

## Abstract

**Background:**

A child suffering from attention deficit hyperactivity disorder (ADHD) faces many difficulties in social as well as academic performances. School teachers’ knowledge and attitude towards ADHD play a vital role in early detection and referral of the child to treatment centers. Few existing reports, however, indicate the alarming rate at which the problem is highly neglected in sub-Saharan Africa. The present study is designed to determine the knowledge, attitude, and factors that affecting elementary school teachers about ADHD.

**Methods:**

An institutional-based cross-sectional study design was conducted in Gondar town and other towns nearby Gondar from February 24 to March 24, 2020. Data were collected through structured self -administered questionnaires using the Knowledge of Attention Deficit Disorders Scale and ADHD-specific attitudes measurement tools. Then, it was entered into Epi-info version 7 and exported to SPSS version 20 for analysis. Bivariable and multivariate logistic regressions were fitted to identify factors associated with the knowledge and attitude of elementary school teachers. Variables having a p-value < 0.05 at 95% CI were considered statistically significant.

**Result:**

Of 636 respondents, about 44.8% (95% CI 41.2, 48.4) and 84.1% (95% CI 81.0, 86.8) of elementary school teachers had good knowledge and a favorable attitude towards ADHD, respectively. Having a diploma and above (AOR = 3.028, 95% CI 1.630–5.625), reading ADHD leaflets (AOR = 2.035, 95% CI 1.391, 2.950) and search ADHD on the internet (AOR = 1.793, 95% CI 1.090, 2.950) were significantly associated with teachers knowledge to ADHD; whereas, working experience in teaching a child with ADHD (AOR = 1.852, 95% CI 1.195–2.87) and watching ADHD on mass media (AOR = 1.72, 95% CI 1.056–2.8) were positively predicts teachers attitude towards ADHD.

**Conclusion:**

the proportion of teachers’ knowledge towards ADHD was low; in contrast, their attitude was relatively satisfactory. Strengthening teachers’ educational upgrading system, frequent and fair distribution of leaflets written to address ADHD, installation of an internet system to the schools, and continuous ADHD awareness creation programs through mass media are highly recommended.

## Background

Attention deficit with or without hyperactivity disorder (ADHD) is one of the most frequently reported neuropsychiatric disorders, especially in school-age children [1]. According to the Fifth Edition of the Diagnostic and Statistical Manual of Mental Disorders (DSM-5), it is characterized by persistent and pervasive problems with inattention and/ or hyperactivity/impulsivity [2]. Commonly, ADHD is diagnosed before the age of 12 years [3]; Likewise, 30–50% of a diagnosed childhood ADHD will be continued until adolescence and adulthood [4]. Even though the exact cause is unknown, genetic, organic, and environmental factors are assumed to be the contributing factors towards the development of ADHD [5].

The global prevalence of ADHD was estimated at 5–7% in children and 2.5% in adults [3, 6]. Even though with this prevalence, teachers knowledge and attitude about ADHD were not satisfactory as evidenced by studies conducted to investigate teachers knowledge such as South Texas (46.49%), Canada (68%), Colombia (48.52%), Pakistan (45.30%), and Egypt (55%), and the attitude in Pakistan(96.2%), and Egypt(55%), respectively [7–13].

ADHD can have an impact on a student’s academic and social performance due to difficulties in maintaining attention, failure to complete tasks, forgetfulness, and excessive non-goal directed physical activities [14]. Moreover, the problem is not limited to in childhood period, it has also an impact on their mental health condition and social wellbeing in adulthood [15], which could be associated with having few or no friends, exercising antisocial behaviors, manifested depressive symptoms, exposed to stress, unable to stick in specific occupations/work, having poor social relationships, using a substance (like alcohol and smoking), exposing unplanned pregnancy, and experiencing multiple car accidents [16–21]. However, since the problem is highly neglected, a majority of children with ADHD remain undiagnosed or do not receive appropriate specialist services in high spite of morbidity [22, 23].

To give timely treatment following early detection, elementary school teachers have been taking the highest place, since most children spend most of their time in schools and interact often with teachers on a daily basis even more than their parents or physicians, suggests that schools play a very important role in the early detection and management of ADHD [3, 24].

Thus, teachers play a central role in early detection and advising parents on managing their children, and implementing classroom and behavioral management strategies [25, 26]. Therefore, it is important for elementary school teachers to have good knowledge and favorable attitudes towards ADHD. However, the evidence in the literature suggests otherwise [26].

Several factors that can affect teachers knowledge and attitude towards ADHD; some of them are, age, sex, marital status, and educational level of the teachers, and searching on the internet about ADHD were factors that affecting the knowledge and attitude of teachers towards ADHD [26–34].

In view of the problem and importance of early detection for effective treatment, different studies should be undertaken to show the prevalence and possible factors that affecting teachers’ knowledge and attitude towards ADHD with its possible recommendation. However, there is a paucity of studies in the nation, Ethiopia, therefore, this study aimed to assess the knowledge and attitude of elementary school teachers about ADHD and its associated factors in Gondar town, Northwest Ethiopia. This could be helpful to design new policies to tackle the problem and make a healthy generation.

## Methods

### Study area, design, and population

An institution-based cross-sectional study design was conducted from February 24 to March 24, 2020, and data were collected from 636 Gondar and nearby town’s public and private elementary school teachers. Gondar town is located in Amhara National Regional State, Ethiopia which is about 727 km Northwest of Addis Ababa (the capital city of Ethiopia). Administratively, Gondar town has a total of 24 kebeles with 13 urban and 11 rural kebeles. Based on reports published by the Central Statistical Agency in 2011, Gondar town has an estimated total population of 254,420 [35]. The town holds several royal castles, including those in Fasil Gebi (the royal enclosure), for which Gondar has been called “Camelot of Africa” [36]. Currently, there are 44 public and 22 private elementary schools in the Gondar and nearby town, which comprise about 3714 elementary school teachers.

### Source and study populations

All elementary school teachers working at public and private elementary schools in Gondar and nearby towns were considered as a source population. Elementary school teachers who are working in the selected Gondar and nearby town’s elementary schools were enrolled as a study population.

### Inclusion and exclusion criteria

All elementary school teachers working at the selected public and private elementary schools in Gondar and nearby towns who were present at the time of data collection were included in the study. Those participants who had a family member with diagnosed ADHD were excluded from the study, since incorporating those teachers having a family member with ADHD might have affected the true magnitude.

### Sample size determination and sampling procedures

The sample size for the first two objectives (knowledge and attitude) was calculated by using single population proportion formula based on the following assumptions 95% confidence level of Z α/_2_ = 1.96, the margin of error = 5%.

$${\text{n}} = \, \left( {{\text{Z }}\alpha /_{{2}} } \right)\,^{{2}} {\text{p }}\left( {{1} - {\text{p}}} \right)/{\text{d}}^{{2}} ,$$

where n = sample size of the population, Z α/_2_ critical value of 95% CI = 1.96, p-proportion (0.564) of knowledge taken from similar previous study’s [34], for attitude 50% was used since there is no study in the country, before.

Then n for Knowledge = (1.96)^2^(0.564) (1–0.564)/0.05^2^ = 378. Thus, by adding 10% for possible non-response rate and 1.5 design effect the total sample size was 624.

n for Attitude = (1.96)^2^(0.5) (1–0.5)/0. 052 = 385. Thus, by adding 10% for possible non-response rate and 1.5 design effect, the total sample size was 636. As a result, we take the highest number i.e., 636 as a final sample size.

**Fig. 1:**
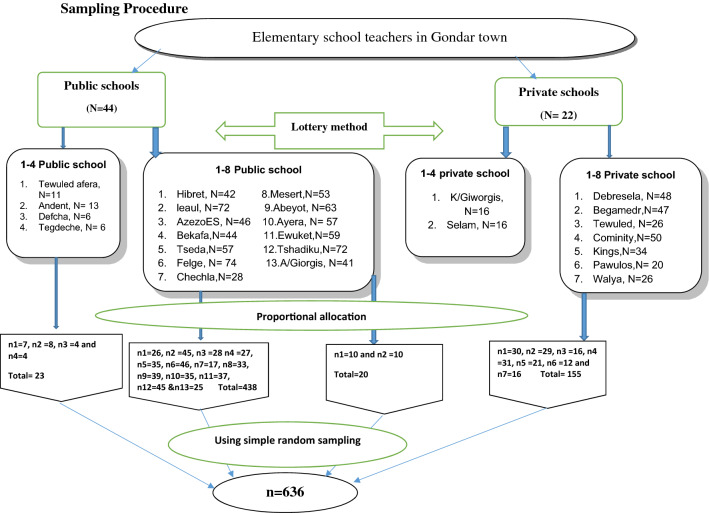
Schematic presentation of the sampling procedure

A multistage sampling procedure was used to identify the study participants. All elementary schools (66 schools) in Gondar town and nearby cities (Tseda and Ambagiorgis) were stratified into public and private elementary schools to allocate the proportion. Then, Forty percent (40%) of the entire school were selected to take an adequate number of schools to represent the source populations [37]. When calculating 40% from public and private elementary schools, seventeen from 44 public (Governmental) and 9 from 22 private schools were included in this study using a lottery method. Among these 26 schools, a total of 1027 teachers were actively working. Then, for the sake of representativeness, Proportional allocation using the formula: ni = (n/N) × Ni where; n = total sample size to be selected, N = total population, Ni = total population of each school, and ni = sample size from each school was used to select the number of teachers participated in the study. Using the sampling frame from the teacher’s staff registration book, 636 study participants were recruited by using a simple random sampling technique (Fig. 1).

### Data collection tools and procedures

The data was collected through a structured self-administered questionnaire adapted from different kinds of literature. It consists of socio-demographic characteristics, characteristics related to the sources of information, knowledge of ADHD, and ADHD-specific attitude.

#### Socio-demographic data

It included age, gender, religion, marital status, type of organization (private and public), years of teaching experience, level of education, grade levels one currently teaches, type of education (general or single subject), and subject currently teaching.

#### Source of information of teachers about ADHD

It included questions such as attend any on-site/off-site training or workshop about ADHD, experience teaching a child who was diagnosed with ADHD, read any books or articles on ADHD, read any leaflet about ADHD, watch any mass media programs about ADHD and searching the internet for getting information about ADHD, with two option response (yes) or (no) format of questions.

#### Knowledge of attention deficit disorders scale (KADDS)

It has 36 items used to assess the knowledge of elementary school teachers. The responses were indicated as ‘true’, ‘false’, and ‘don’t know’ responses. It has contained three subscales: (a) general information of teachers (15 items), (b) symptoms and diagnosis (9 items), and (C) knowledge about treatment (12 items) Accordingly, the passing score of this scale was 50% [29] on each subscale as well as the total scale. The reliability test was performed for KADDS questionnaires using Cronbach’s alpha and initially, the value was 0.79.

#### ADHD-specific attitudes (SASA)

A 5-point Likert-type SASA scale was used to measure teacher’s attitudes regarding ADHD; it was ranged from ‘strongly disagreed’, ‘disagreed’, ‘neutral’, ‘agreed’, to ‘strongly agreed’. During analysis, several items were again reversing reversed scores to ensure the conscious completion of the questionnaires. The total score was calculated by summing up and converted into a percentage score. Accordingly, teachers’ who scored ≥ 60.0% from the aforementioned attitude questions were considered as having a favorable attitude towards ADHD [38].

### Data quality control

To maintain the quality of the data, the tool was carefully adapted from different works of literature in English, then it was translated to Amharic and again translated back to English by language experts working in University of Gondar, language and Literature department with mental health professionals to check its consistency. The content of the questionnaire was reviewed by psychiatrists. One day of training about the objectives, significance, and variables of the study was given to four data collectors and two supervisors by the principal investigator before the actual data collection. A pretest was conducted in about 5% of the samples out of selected schools (Keye Anba, Azezo Teklehaimanot, and Desalegne elementary schools). The data collection process was closely monitored by the principal investigator for the completeness of the data. Each questionnaire was checked regularly for completeness to identify the gaps and act immediately.

### Data processing and analysis

After checking the completeness and consistency, the data was entered into Epi-info version 7 and then exported to SPSS version 20 for cleaning, coding, and analysis. Descriptive statistics were carried out and summarized by using texts, tables, charts, and graphs. The prevalence of knowledge and attitude towards ADHD was computed and reported as percentages. To identify the major determinant factors, a binary logistic regression model was employed and Multivariate analysis was used to identify the confounders. All variables were entered into a multivariable logistic regression to identify factors that have a statistically significant association. Thus, variables having a p-value of ≤ 0.05 were considered significant. Adjusted odds ratio (AOR) with a 95% confidence interval was used to show the strength of association. The model’s fitness goodness-of-fit was checked by Hosmer and Lemeshow test [39].

## Result

### Socio-demographic characteristics

With a response rate of 100%, 636 respondents were enrolled in the study. Among them, more than half (59%) were female participants and about one-third (37.6%) of teachers were between the age of 20–30 years old with a mean of 36.77 (SD ± 10.72). Moreover, a majority (66.7%) of the participants were married, just half (50.9%) had diploma holders, and about half (50.2%) of teachers were teaching grades 5 to 8. Of the participants, below half (44.2%) of teachers had greater than fifteen years of work experience. Likewise, significant proportions (85.1%) of the participants were delivering only one type of subject/course (Table 1).

**Table 1:** Socio-demographic and academic characteristics of knowledge and attitude towards ADHD among elementary school teachers in Gondar town, Ethiopia, May 2020 (n = 636)

Variable	Frequency	Percent
Age		
20–30	239	37.6
31–40	223	35.1
41–50	74	11.6
> 51	100	15.7
Gender		
Male	261	41
Female	375	59
Religion		
Orthodox	582	91.5
Muslim	26	4.1
Catholics	6	0.9
Protestant	22	3.5
Marital status		
Single	160	25.2
Married	424	66.7
Divorced	43	6.8
Widowed	9	1.4
Type of organization		
Private	174	27.4
Public	462	72.6
Work experience		
< 5 years	118	18.6
5–9 years	113	17.8
10–14 years	124	19.5
> 15 years	281	44.2
Educational status		
Certificate	68	10.7
Diploma	324	50.9
Degree and above	244	38.4
Teaching grade level		
Kindergarten (KG) 1–3	35	5.5
1–4	282	44.3
5–8	319	50.2
Classes you currently teach		
General education	95	14.9
Single education	541	85.1
Subject type		
Aesthetics and/or physical education	56	8.8
Natural science or technology	193	30.3
Social science	233	36.6
All subject	62	9.7
Special need	92	14.5

### Source of information regarding ADHD

Among the respondents, 495 (77.8%) of them did not take any training, workshop, and/or courses regarding ADHD. Nearly two-thirds of the respondents (63.8%) had experience of teaching students with ADHD. About 236 (37.1%), 222 (36.2%), 186 (29.2%), and 87(13.7%) of the respondents were got received information from mass media, books, and articles, reading the pamphlet and searching from the internet, respectively (Table 2).

**Table 2:** Source of information regarding knowledge and attitude towards ADHD among elementary school teachers’ in Gondar town, Ethiopia, May 2020 (n  =  636)

Variable	Frequency	%
Training and workshop about ADHD		
No	495	77.8
Yes	141	22.2
Experience in teaching to a child with ADHD		
No	230	36.2
Yes	406	63.8
Read any books and article		
No	414	63.8
Yes	222	36.2
Read any leaflet		
No	450	70.8
Yes	186	29.2
Watch any mass media		
No	400	62.9
Yes	236	37.1
Search on the internet		
No	549	86.3
Yes	87	13.7

### Knowledge of teachers’ towards ADHD (KADDS)

**Fig. 2:**
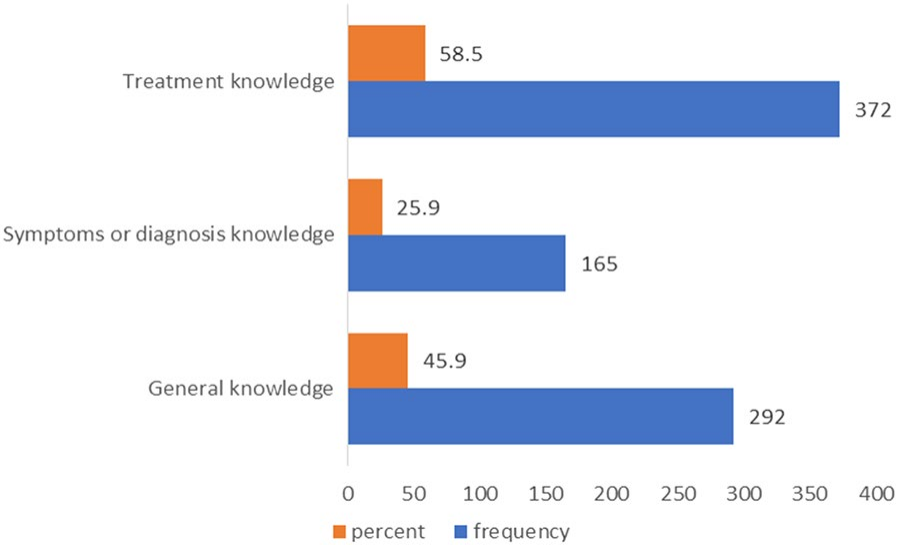
The subscales of knowledge questions used to assess elementary school teachers’ knowledge about ADHD (n = 636)

Of the total respondents, below half (44.8% [95% CI 41.2, 48.4]) of them had good knowledge about ADHD. Specifically, among the subscale of knowledge questions, more than half (58.5%) had good knowledge regarding the treatment of ADHD (Fig. 2).

### An attitude of elementary school teachers towards ADHD

Regarding the attitude of elementary school teachers about ADHD, 84.1% [95% CI 81.0, 86.8] of the respondents had a favorable attitude towards ADHD.

### Factors associated with knowledge of elementary school teachers towards ADHD

The bivariable logistic regression analysis showed that educational status, reading leaflets, watching mass media, and searching on the internet about ADHD were significantly associated with having a good knowledge of ADHD in elementary school teachers. Finally, after running a multivariate analysis among those variables having a p-value of ≤ 0.05 in the bivariable analysis, educational status (diploma, degree, and above), reading ADHD leaflet, and searching on the internet remain significant.

The odds of having good knowledge was 3.0 times higher among elementary school teachers who had diploma (AOR = 3.028, 95% CI 1.630, 5.625) and 3.1 times higher among elementary school teachers who had a degree and above educational status (AOR = 3.134, 95% CI 1.664, 5.900) as compared with elementary school teachers who had a certificate.

The odds of having good knowledge was about two times higher among elementary school teachers having experience of reading leaflet as compared to their counterparts (AOR = 2.035, 95% CI 1.391, 2.950). The likelihood of having good knowledge was 1.79 times higher among elementary school teachers having experience of internet searching as compared to their counterparts (AOR = 1.793, 95% CI 1.090, 2.950) (Table 3).

**Table 3:** Factors associated with knowledge towards ADHD among elementary school teachers’ in Gondar town, Ethiopia, May 2020 (n = 636)

Variables	Knowledge about ADHD		Crude OR (95% CI)	Adjusted OR (95% CI)
	Good	Poor		
Educational status				
Certificate Diploma Degree and above	16 154 115	52 170 129	1 2.944 (1.614, 5.371)** 2.897 (1.568, 5.354)	1 3.028 (1.630, 5.625)** 3.134 (1.664, 5.900)**
Read any leaflet about ADHD				
No Yes	172 113	278 73	1 2.502 (1.762, 3.552)**	1 2.035 (1.391, 2.950)**
Watch any mass media				
No Yes	84 201	152 199	1 1.828 (1.313, 2.544)	1 1.394 (0.975, 1.993)
Search on the internet				
No Yes	231 54	318 33	1 2.253 (1.415, 3.586)*	1 1.793 (1.090, 2.950)*

Reference **p < 0.001, *p < 0.05

### Factors associated with an attitude of elementary school teachers towards ADHD

The multivariate logistic regression analysis, having teaching experience of students with ADHD, and watching mass media were significantly associated with the attitude of elementary school teachers.

The odds of having a favorable attitude were nearly 1.85 times higher among elementary school teachers having teaching experience to a child with ADHD as compared to their counterparts (AOR = 1.852, 95% CI 1.195, 2. 87). The likelihood of having a favorable attitude was 1.7times higher among elementary school teachers watching any mass media than their counterparts (AOR = 1.72, 95% CI 1.056, 2.8) (Table 4).

**Table 4:** Factors associated with an attitude towards ADHD among elementary school teachers’ in Gondar town, Ethiopia, May 2020. (n = 636)

Variables	Attitude towards ADHD		Crude OR (95% CI)	Adjusted OR (95% CI)
	Favorable	Unfavorable		
Experience in teaching to a child with ADHD				
No Yes	181	49	1 1.84 (1.2, 2.83)	1 1.85 (1.195, 2.87)*
	354	52		
Taking ADHD training/workshop				
No Yes	422	73	1 0.69 (0.431, 1.13)	1 0.63 (0.38, 1.05)
	113	28		
Read any books or article about ADHD				
No Yes	353	61	1 0.78 (0.50, 1.21)	1 0.64 (0.39, 1.05)
	182	40		
Watch any mass media				
No Yes	191	45	1 1.44 (0.94, 2.22)	1 1.72 (1.05, 2.8)*
	344	56		
Marital status				
Single Married	186	26	1.53 (0.95, 2.48) 1	1.56 (0.96, 2.55) 1
	349	75		

Reference *p < 0.001

## Discussion

This study was conducted aimed to ascertain the knowledge, attitude, and associated factors towards ADHD among elementary school teachers in Gondar and nearby towns, Ethiopia. In the current study, the proportion of having knowledge bout ADHD among elementary school teachers was 44.8% [95% CI 41.2, 48.4], which indicated that there is a knowledge gap in this population about the problem, therefore, a comprehensive and well-designed strategy will be mandatory for making a timely solution to minimize the possibility of delayed diagnosis of ADHD and classroom mistreatment [40–42].

This study finding was in line with a study conducted in Cape Town (South Africa) [43]. Nonetheless, the finding was far higher and higher than the studies done in Saudi Arabia at 11%, Thailand at 19.4%, Egypt at 23.9%, Nepal city (Asia) 24.2%, and India 40% [13, 29, 44, 45]. Even if, the studies were used a cross-sectional design the same as our study, the observed discrepancy might be due to the study design such as sample size and sampling method (random and convenient sampling technique was used in Saudi Arabia and Egypt study’s [13, 46]) as well as the difference in cultural characteristics too [13, 29, 44, 45].

On the other hand, the current finding was lower than studies conducted in Canada 68%, Colombia 48.52%, Saudi Arabia 58.9%, another Saudi Arabia study 72%, India 49%, and South Africa 78%, [8, 9, 12, 27, 28, 33, 34, 46]. The discrepancy could be attributed to educational curricula that were used to teach teachers and the highest educational level attained was observed [8, 9, 12, 27, 28, 33, 34, 46].

In the current study, having good knowledge about ADHD was significantly associated with participants’ educational level of diploma and degree than those having a certificate. The same has been reported by others [28, 32]. It is likely that the participants’ highest educational level attained the capacity of acquiring knowledge towards different issues including ADHD will be increased [47]. Therefore, to increase the understanding of ADHD and the implementation of behavioral strategies, teachers holding lower educational qualifications could be encouraged to seek training regarding ADHD.

This study came up with having good knowledge was statistically significant with teachers reading ADHD leaflets. This might be due to the content of leaflets is minimal with a brief description of the information and easy to handle [48, 49]. Hence, reading leaflets is one of the effective ways of promoting and educating people about health.

The study evidenced that the higher odds of having Good knowledge were observed among participants of the internet user. The same has been reported [34]. This might be due to the fact that using the internet is a good way of acquiring knowledge and new ways of learning environment for most teachers since it is rapid, cost-effective, get reliable information and publications which enhance knowledge [50–53]. However, in Ethiopia, only 17.8% of the population had internet accesses [54]; moreover, this study also evidenced that more than three-fourth (86.3%) of respondents did not use the internet to read about ADHD which indicates that internet accesses is a commonly faced problem among elementary school teachers.

An important finding observed in this study is participant’s attitudes about ADHD, the study revealed that 84.1% of elementary school teachers were detected to have a favorable attitude, which indicated that the existing attitude towards ADHD is good. Even though, to address the issues at large for covering the rest of the participants having an unfavorable attitude, a well-designed strategy and policy still needed. The current finding was higher than a study done in Iran, South Africa, Egypt, and Nigeria [12, 13, 46, 55]. The discrepancy might be due to the difference in sample size (n-120, 360, and 250) and sampling method (random sampling technique) was used in Iran, Egypt, and Nigeria studies and the difference in cultural characteristics too [12, 13, 46, 55]. Nonetheless, the result of this study was lower than a study conducted in Pakistan (92.2%) [10]. The observed discrepancy might be due to the tool difference that measures ADHD which is Conner’s teacher’s rating scale [10].

Regarding factors associated with the participant's attitude towards ADHD; The odds of having a favorable attitude was higher among elementary school teachers who had a teaching experience of a child with ADHD. This supported finding is in line with a study conducted in Saudi Arabia [28]. This could be explained by Having experience might be the most common source of knowing the problem to some extent and knowledge is found upon the accumulation of information through either experience or education [47, 56].

Finally, this study found that the likelihood of having a favorable attitude was higher among elementary school teachers watching any mass media programs. This might be due to the mass media may play an important role in sharing health information and increasing awareness about health since media not only spread awareness but also report new knowledge, scientific information, and research findings to the public which has ultimately helped in the change of attitude of the audience for achieving better health [57, 58].

Doing the study in this overlooked area of mental health (ADHD) especially in the country with a paucity of researches was the good looks of the study and the findings could alarm the responsible body’s pleasing to the eye on Childhood mental health. Nonetheless, the fact that the study was a cross-sectional survey, it is difficult to derive causal relation. Likewise, it would have been more novel if it was conducting a training and re-administer the survey to teachers to see if attitudes/knowledge of ADHD has changed. Moreover, it would have been more soundable if it incorporates the socio-economic status of the teachers as this may possibly also have an impact on the knowledge and attitudes of ADHD.

## Conclusion

The proportion of elementary school teachers who had good knowledge of ADHD was low, whereas, the proportion of teachers who had a favorable attitude towards ADHD was relatively satisfactory. Educational status, reading the leaflet, and searching on the internet were variables significantly associated with the knowledge of teachers about ADHD. On the other hand, the experience of teaching children with ADHD and watching mass media were significantly associated with the attitude of elementary school teachers regarding ADHD. Therefore, to enhance teachers’ understanding of ADHD and implementation of behavioral strategies in the classroom, teachers with lower educational status like certificates could be encouraged to seek training regarding ADHD. Moreover, policymakers should consider the incorporation of ADHD and other childhood mental health issues in primary school teachers’ curriculum. Likewise, Frequent and fair distribution of leaflets that are talking about ADHD, installation of the internet to the schools considering easy accessibility, and continuous ADHD awareness creation through mass media are highly recommended.

## Data Availability

All relevant data are available within the manuscript.

## Abbreviations

ADHD: Attention deficit-hyperactivity disorder
AOR: Adjusted odds ratio
DSM: Diagnostic and statistical manual
KADDS: Knowledge of Attention Deficit Disorders Scale
SASA: ADHD-specific attitudes
SPSS: Statistical package for social science
